# Removal of sequencing adapter contamination improves microbial genome databases

**DOI:** 10.1186/s12864-024-10956-1

**Published:** 2024-11-04

**Authors:** Andrew H. Moeller, Brian A. Dillard, Samantha L. Goldman, Madalena V. F. Real, Daniel D. Sprockett

**Affiliations:** 1 Department of Ecology and Evolutionary Biology, Princeton University, Princeton, NJ 08540 USA; 2 Department of Ecology and Evolutionary Biology, Cornell University, Ithaca, NY 14853 USA

**Keywords:** Bacterial genomes, Metagenome, Microbiota, Symbiosis, Microbial ecology

## Abstract

Advances in assembling microbial genomes have led to growth of reference genome databases, which have been transformative for applied and basic microbiome research. Here we show that published microbial genome databases from humans, mice, cows, pigs, fish, honeybees, and marine environments contain significant sequencing-adapter contamination that systematically reduces assembly accuracy and contiguousness. By removing the adapter-contaminated ends of contiguous sequences and reassembling MGnify reference genomes, we improve the quality of assemblies in these databases.

**Supplementary Information:**

The online version contains supplementary material available at 10.1186/s12864-024-10956-1.

## Background

Recent work has generated unprecedented numbers of microbial genome sequences from the microbiomes of eukaryotic hosts and free-living habitats [1–3]. Databases of thousands of microbial-genome assemblies from isolates and metagenomes (i.e., metagenome-assembled genomes—MAGs) are now available from humans [4], mice [5], cows [6], pigs [7], chicken [8], fish [3], and honeybees [9] as well as marine environments [3]. These resources are enabling previously intractable functional and evolutionary studies of microbiomes [10, 11].

The large size of recently published microbial genome databases requires automated approaches for inspection and quality control of individual assemblies [12]. Automated tools for detecting chimeras and measuring strain heterogeneity, completeness, contamination, and contiguousness have been developed [13–17]. Sequencing adapter contamination is a known issue with assembly of reads from commonly used technologies (e.g., Illumina) in which sequences from adapters used during the sequencing process are erroneously incorporated into assemblies [18–22]. To mitigate this issue, studies typically remove adapter sequences from reads prior to assembly [10], such that adapter contamination is not expected to be prevalent in these databases. For instance, studies from which reference-genome databases in the MGnify repository were derived all reported efforts to clean sequence reads of adapter contamination before assembly [3–9]. However, the incidence of adapter contamination in these databases has not been investigated. Here, we demonstrate a significant extent of sequencing adapter contamination in MGnify microbial genome databases—and develop an approach for the elimination of this contamination—with the goal of improving the accuracy, contiguousness, and utility of these resources for future studies.

## Results

### Significant evidence of adapter contamination in MGnify databases

To evaluate the extent to which assemblies contain sequencing-adapter contamination, we calculated the baseline rate at which adapter sequences are expected to be observed by chance in a genome assembly of a given length. Illumina sequencing of TruSeq libraries employs the 12-base universal adapter sequence ‘AGATCGGAAGAG’, which has a $\frac{1}{{4}^{12}}$ probability of being observed by chance in a biological sequence of 12 bases in length, assuming equal probability of each nucleotide at each site and independence among sites. Thus, ‘λ’, the number of adapter sequences expected to be observed by chance in an assembly of ‘*X*’ length containing ‘*y*’ contiguous sequences (contigs) is given by Eq. 1. The integer ‘11’ in Eq. 1 reflects that the last 11 bases in each contig cannot be the start of a 12-base adapter sequence.

1 $$\uplambda =\frac{\left(X-11y\right)}{{4}^{12}}$$

The probability of observing greater than or equal to a specific number of ‘*k*’ sequences in a sequence of ‘*X*’ sites can be calculated using the Poisson cumulative distribution function (Online Methods):

2 $$\text{ Pr}\left(O\ge k\right)=1-{e}^{-\uplambda }\sum_{j=0}^{k-1}\frac{{\uplambda }^{j}}{j!}$$

Equations 1 and 2 can be used to calculate a *p*-value corresponding to the probability of observing by chance *k* or more adapter sequences, providing a test for significant levels of adapter contamination in an assembly given its length and number of contigs.

**Fig. 1:**
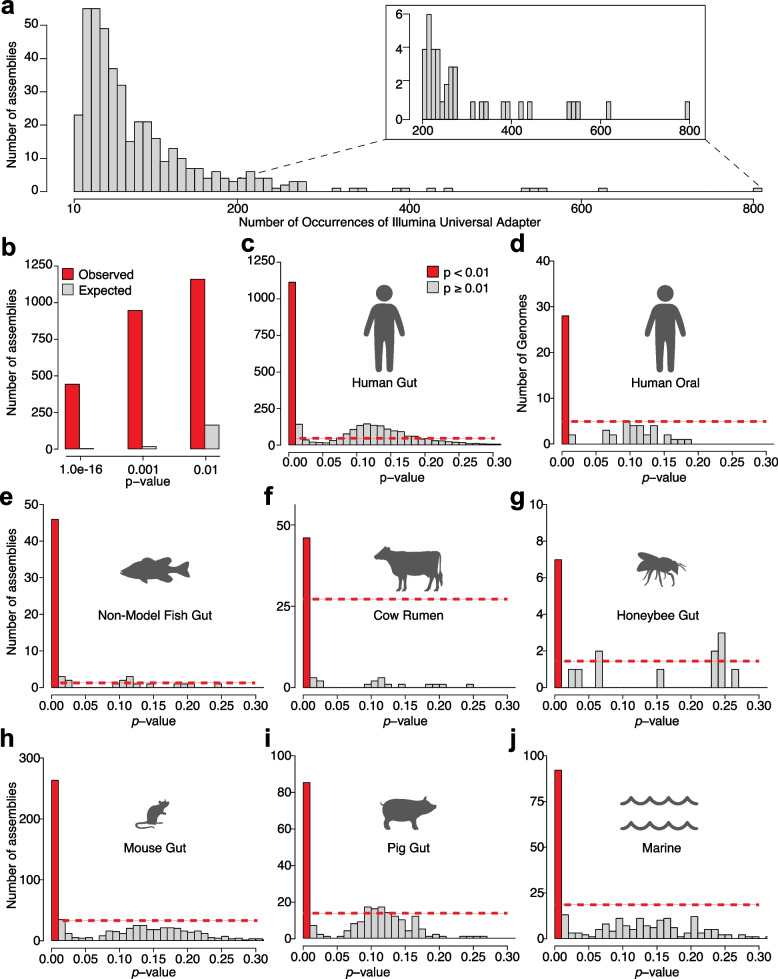
Significant enrichment of Illumina adapter sequences in published microbial genome databases. **a** Histogram shows the number of assemblies in all databases containing 10 or more exact matches to the Illumina universal adapter sequence or its reverse complement. Of the 15,657 species reference genome assemblies, the number of assemblies expected to contain 10 or more exact matches by chance was ~ 1.57e-12, i.e., ~ 0. **b** Bar plot shows the number of assemblies displaying significant evidence of adapter enrichment at three *p*-value thresholds. Expected number of assemblies is shown for each threshold. **c–j** Histograms show the number of assemblies in individual databases for specific ranges of *p*-values. In (**c–j**), Red bars indicate the number of assemblies for which *p*-values were < 0.01. Dashed red lines indicate the number of assemblies expected to display *p*-values of < 0.01 by chance (i.e., ~ 1% of assemblies in each database)

Using this approach, we tested for adapter enrichment in every microbial species’ reference genome assembly in microbial genome databases from environments represented in MGnify [3], including ‘human gut’, ‘human oral’, ‘human vaginal’, ‘mouse gut’, ‘pig gut’, ‘cow rumen’, ‘honeybee gut’, ‘non-model fish gut’, ‘zebrafish fecal’, ‘chicken gut’, and ‘marine’. The number of adapter sequences observed per assembly (including both forward and reverse complement orientations of the adapter sequence) ranged from 0 to 805, with a *Paenibacillus lactis* assembly from the human gut (accession MGYG000003402) displaying the most adapter sequences. A histogram of assemblies containing 10 or more adapter sequences is shown in Fig. 1a. Of the 15,657 species reference genome assemblies in all MGnify databases, only ~ 157, ~ 15.7, and ~ 1.57e-12 assemblies were expected to be observed by chance at the thresholds of *p*-value < 0.01, 0.001, and 1e-16, respectively. In contrast, 1110, 888, and 433 assemblies contained significant enrichment of adapters at these *p*-value thresholds, respectively (Fig. 1). An enrichment of assemblies displaying significant *p*-values was also observed within individual databases (Fig. 1c–j). A total of 1020 assemblies displayed significant evidence of adapter contamination after false-discovery rate (FDR) correction for testing of multiple assemblies, and enrichment of adapters was evident in assemblies derived from isolates as well as MAGs (Table S1). These results show significant adapter contamination in microbial genome assemblies in these reference databases.

### Concentration of adapter sequences in extremities of contigs

**Fig. 2:**
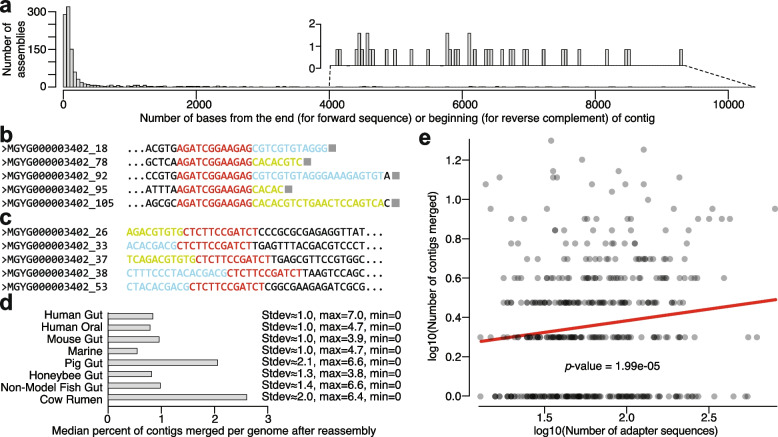
Adapter contamination is concentrated at the beginnings and ends of contigs, and its removal improves assembly contiguousness. **a** Histogram shows the concentration of Illumina universal adapter sequences near the extremities of contigs in the genomes showing significant evidence of adapter contamination (*p*-value < 0.01). Mean distances in bases from beginnings or ends of contigs were calculated for adapter sequences and reverse complements of adapter sequences, respectively. **b** DNA sequences show five examples of contamination by Illumina adapters (red sequences) at the ends of contigs (grey squares) in *Paenibacillus lactis* assembly MGYG000003402 from the human gut. **c** DNA sequences show five examples of contamination by the reverse complement of Illumina adapters (red sequences) at the beginnings of contigs in assembly MGYG000003402. In (**b**) and (**c**) blue and yellow sequences correspond to forward- and reverse-specific adapter sequences, respectively, adjacent to the universal adapter sequence. **d** Barplot shows for each database the per-assembly average number of contigs merged with other contigs after the removal of adapter contamination and reassembly (of the 1110 contaminated assemblies at *p*-value < 0.01). **e** Scatterplot shows the positive relationship between the number of adapter sequences present in assemblies showing the strongest evidence of contamination (FDR-corrected *p*-value < 1e-16) (x-axis) and the number of contigs that were able to be merged by reassembly after adapter contamination removal (y-axis). Red line shows best-fit regression of log transformed values (transformation was made to reduce heteroscedasticity). The *p*-value was calculated from a generalized linear model with Poisson-distributed errors for count data

Adapter sequences were concentrated at the ends of contigs, and the reverse complements of adapter sequences were concentrated at the beginnings of contigs (Table S1) (Fig. 2a). For example, in the *Paenibacillus lactis* assembly containing 319 adapter sequences in the forward orientation, the average distance of the end of the adapter sequence to the end of the contig in which it was found was only ~ 10 bases, with a maximum distance of 74 bases and a minimum distance of 0 bases (i.e., the last base in the contig was the last base of the adapter sequence), despite the average length of contigs in this assembly being ~ 2900 bases (Fig. 2b, Table S1). Conversely, the reverse complements of the adapter sequence were clustered near the beginnings of contigs in this assembly (Fig. 2c, Table S1). Instances of the adapter sequence were also adjacent to portions of known forward- or reverse-specific adapter sequences (‘CACACGTCTGAACTCCAGTCA’ and ‘CGTCGTGTAGGGAAAGAGTGT’, respectively) or their reverse complements (Fig. 2b, c). Concentration of contamination at the beginning or ends of contigs was also observed in the other adapter-contaminated assemblies (Table S1).

### Removing adapter contamination and reassembling contigs improves assembly contiguousness

Previous work has shown that adapter contamination can inhibit the merging of contigs during the assembly process [21, 22]. Because the clustering of adapter contamination at the beginnings or ends of contigs is consistent with the possibility that adapter contamination broke contigs during the assembly process, we reasoned that the contiguousness of assemblies might be improved by trimming the ends of contaminated contigs and attempting to stitch together the trimmed contigs. We trimmed the last (or first) 450 bases of every contig containing an adapter sequence within 300 bases of the end (or beginning) of the contig—thereby removing the adapter sequences and their flanking regions—and reassembled the trimmed contigs of every species reference genome assembly. Trimming 450 bases yielded the highest average increase in the contiguousness of assemblies, as measured by N50, compared to other trimming lengths tested (Online Methods). Reassembly following removal of adapter contamination increased N50 for 327 of the 1110 assemblies containing significant evidence of adapter sequences at the *p*-value < 0.01 threshold, with an average increase of 917 bases and a maximum increase of 10,258 bases (Table S2). These values correspond to improvements in N50 of up to 20% for individual assemblies.

Contiguousness of assemblies was improved in each individual database (Fig. 2d). On average, ~ 2 contigs per assembly, corresponding to ~ 0.8% of all contigs, were merged with other contigs after removing adapter contamination. For 211 assemblies, 10 or more contigs were merged, with a maximum of 54 contigs merged for a single assembly. Moreover, we observed a positive relationship between the number of adapter sequences present in an assembly and the number of contigs that were assembled with other contigs after removing adapter contamination (Fig. 2e) (generalized linear model with Poisson-distributed errors for count data, *p*-value = 1.99e-5). These results further indicate that adapter contamination negatively affects assembly contiguousness and that these negative effects can be mitigated by removal of adapter contamination and reassembly.

## Discussion

In this study, we identified and remedied widespread sequencing-adapter contamination in published MGnify microbial genome databases. Corrected assemblies generated by this study (trimmed assemblies and reassemblies) are available at https://zenodo.org/records/10547057. Scripts for detecting and assessing the extent of adapter contamination in assemblies, removing the ends of adapter-contaminated contigs, and reassembling trimmed contigs are available at github.com/CUMoellerLab/MalAdapter. The increased contiguousness of assemblies reported here may improve their utility for studies focused on structural features of microbial genomes such as gene order, operons, accessory chromosomes, and repetitive elements. Moreover, removing adapter sequences increases assembly accuracy, which may improve the utility of assemblies for any future study.

## Methods

### Data sources

Genome assemblies for this study were downloaded from the MGnify [3] ftp site at https://ftp.ebi.ac.uk/pub/databases/. Most recent versions of each database were used as follows: ‘chicken-gut’ = v1.0.1, ‘cow-rumen’ = v1.0.1, ‘honeybee-gut’ = v1.0.1, ‘human-gut’ = v2.0.2, ‘human-oral’ = v1.0.1, ‘human-vaginal’ = v1.0, ‘marine’ = v1.0, ‘mouse-gut’ = v1.0, ‘non-model-fish-gut’ = v2.0, ‘pig-gut’ = v1.0, ‘zebrafish-fecal’ = v1.0.

### Derivation of expectations and probabilities and correction for multiple testing

The term ‘k minus 1’ in Eq. 2 allows the calculation of the probability of observing greater than or equal to ‘k’ adapter sequences. Because multiple genomes were tested, corrections to *p*-values were made based on a false-discovery rate of 0.1, yielding q-values for each assembly. The results were qualitatively robust to the choice of q-value threshold: significant evidence of adapter contamination was observed at q-value thesholds as low as 1e-16.

### Identification and removal of adapter sequences

Illumina universal adapter sequences (‘AGATCGGAAGAG’) and their reverse complements (‘CTCTTCCGATCT’) were identified and counted in assemblies using custom bash scripts available at github.com/CUMoellerLab/MalAdapter. When adapter sequences were detected within the first or last 300 bases of a contig, the first or last 450 of the contig was removed, respectively. Testing trimming lengths of 150, 250, 350, 550, and 650 bases supported that 450 bases yielded the highest mean improvement to N50, although all choices of trimming length yielded qualitatively similar results (mean N50 increase was within 6.2% regardless of trimming length chosen).

### Reassembly of contigs and calculation of N50

To reassemble trimmed contigs after the removal of adapter contamination at the ends of contigs, we employed CAP3 [23] using the following settings: -z 1 -y 6 -f 2 -p 99. These choices enabled the stitching together of the ends of contigs with perfectly overlapping and identical regions. N50 was calculated using custom bash scripts available at github.com/CUMoellerLab/MalAdapter.

## Supplementary Information

Supplementary Material 1: Table S1. Results of analyses of adapter enrichment.

Supplementary Material 2: Table S2. Differences in N50 resulting from adapter removal. Table shows MAGs display increases in N50 values after adapter removal compared to before adapter removal.

## Data Availability

All data used in this study were generated by previous work and are publicly available at https://www.ebi.ac.uk/metagenomics.
